# Central Cell in Flowering Plants: Specification, Signaling, and Evolution

**DOI:** 10.3389/fpls.2020.590307

**Published:** 2020-10-21

**Authors:** Hong-Ju Li, Wei-Cai Yang

**Affiliations:** ^1^State Key Laboratory of Molecular Developmental Biology, Institute of Genetics and Developmental Biology, Chinese Academy of Sciences, Beijing, China; ^2^College of Advanced Agricultural Sciences, University of Chinese Academy of Sciences, Beijing, China

**Keywords:** central cell, double fertilization, flowering plants, gymnosperm, cell specification, cell-cell communication

## Abstract

During the reproduction of animals and lower plants, one sperm cell usually outcompetes the rivals to fertilize a single egg cell. But in flowering plants, two sperm cells fertilize the two adjacent dimorphic female gametes, the egg and central cell, respectively, to initiate the embryo and endosperm within a seed. The endosperm nourishes the embryo development and is also the major source of nutrition in cereals for humankind. Central cell as one of the key innovations of flowering plants is the biggest cell in the multicellular haploid female gametophyte (embryo sac). The embryo sac differentiates from the meiotic products through successive events of nuclear divisions, cellularization, and cell specification. Nowadays, accumulating lines of evidence are raveling multiple roles of the central cell rather than only the endosperm precursor. In this review, we summarize the current understanding on its cell fate specification, intercellular communication, and evolution. We also highlight some key unsolved questions for the further studies in this field.

## Introduction

Unlike that in animals where the haploid spores generated by meiosis directly differentiate into functional gametes, in plants the haploid spores undergo additional mitosis to produce multicellular gametophytes. In lower plants, like the Bryophyte, gametophytes are dominant of their life cycles and unusually free-living, whereas in seed plants (gymnosperms and angiosperms), the gametophytes are structurally reduced and develop within the sporophytic sexual organs. In flowering plants (angiosperms), the female gametophytes are developmentally reduced to a miniature structure with only a few cells embedded within layers of sporophytic ovular tissues. In contrast to the gametophyte-dominant species and sporophyte-dominant gymnosperms, the emergence of an additional female gamete, the central cell, is a critical innovation of sexual reproduction and a hallmark of angiosperms. Fertilization of both the egg and the central cell, known as double fertilization, produces the embryo and endosperm, respectively, within the seed coat. Besides acting as the endosperm precursor, the central cell also undertakes important roles during embryo sac development and function. This review outlines recent advances in our understanding of the central cell with focuses on cell specification, cell-to-cell communication, and evolution.

## Types of Embryo Sac

**FIGURE 1:**
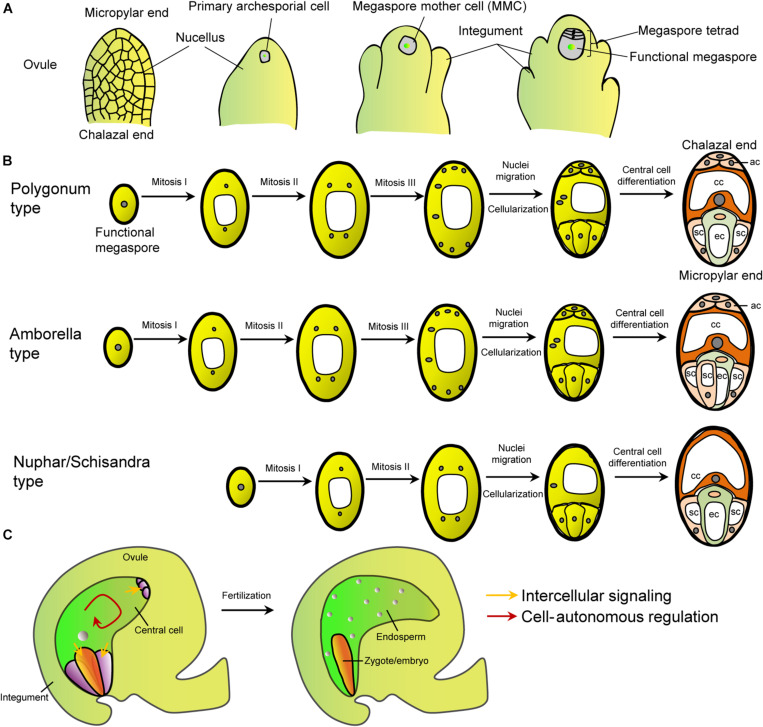
The female gametophyte patterns and intercellular signaling between central cell and other cells. **(A)** Schematic diagram of the megasporogensis. **(B)** Schematic diagram of three female gametophyte types mentioned in the text. The Polygonum type is exhibited by most (>70%) flowering plants, including Brassicaceae, Gramineae, Malvaceae, Leguminoseae, and Solanaceae (Yadegari and Drews, 2004). The Amborella and Nuphar/Schisandra types are the primitive ones. Other types can be referred to reviews by Yadegari and Drews (2004), Schmid et al. (2015), and Gonzalez et al. (2019). White shapes within the cells represent vacuoles. **(C)** Schematic diagram of the female gametophyte within an Arabidopsis ovule and embryo and endosperm after fertilization. Arrows, the intercellular and cell-autonomous signaling.

In flowering plants like Arabidopsis, a single megaspore mother cell (MMC) was initiated at the tip of each nucellus (Yadegari and Drews, 2004; Shi and Yang, 2011; Yang et al., 2010; Lora et al., 2019). Through meiosis, the MMC produces four haploid megaspores, of which only one becomes the functional megaspore, the other three are degenerated (Figure 1A). The functional megaspore undergoes three rounds of nuclear mitosis to form a syncytial female gametophyte with eight nuclei. Subsequently, the syncytial female gametophyte undergoes simultaneous cytokinesis to form a typical eight-nucleated, seven-celled embryo sac (Figure 1B; Christensen et al., 1997; Shi and Yang, 2011; Yang et al., 2010). Finally, cell fate specification and maturation take place to generate the four cell types within the functional female gametophyte: two synergid cells and an egg cell at the micropyle end, a diploid central cell and three antipodal cells at the chalazal end that connects tightly with the maternal tissues (Figure 1B). This developmental pattern is known as monosporic Polygonum-type that exists in most angiosperms. In the model plant Arabidopsis, the three antipodal cells are short-lived, while in monocot, they proliferate and participate in the endosperm development. As an exception, the basal angiosperm *Amborella trichopoda*, a single extant species, forms a unique Amborella-type embryo sac that contains three synergid cells due to an extra cell division of one of the micropylar cells (Figure 1B; Friedman, 2006; Friedman and Ryerson, 2009). Another group of basal flowering plants, Nymphaeales (including Hydatellaceae) and Austrobaileyales, exhibits the Nuphar/Schisandra-type embryo sac that is four-celled with a haploid central cell and without antipodals (Figure 1B; Baroux et al., 2002; Friedman, 2008; Povilus et al., 2015; Zini et al., 2016). Other types of embryo sacs also exist in a number of angiosperm taxa in nature, such as the bisporic and tetrasporic types (Friedman et al., 2008; Schmid et al., 2015). All the different patterns appear to be modular with the micropylar egg-apparatus module and the chalazal module across species (Friedman and Williams, 2004). It is unknown whether the variation of female gametophyte structure among plant taxa has any adaptive significance. It has been suggested that developmental lability at the earliest stage of angiosperm evolution may lead to this variation (Friedman, 2006). Given the diverse structure, the molecular determination of the central cell may vary from taxa to taxa. This makes the generalization of the regulation of embryo sac development and its evolutionary origin difficult. Nevertheless, ubiquity of the four-cell types indicates that a few conserved factors might be enough to orchestrate the structural organization and cell fate determination. So far, our understanding on molecular regulation of female gametophyte development is mostly from the model plant Arabidopsis and a few crop species.

During the female gametophyte development, positional cues, hormones, and coordinated cell-to-cell communication have been proposed to orchestrate the establishment of embryo sac polarity and development (Kägi and Groß-Hardt, 2007; Pagnussat et al., 2007, 2009; Moll et al., 2008; Sundaresan and Alandete-Saez, 2010; Kirioukhova et al., 2011; Lituiev et al., 2013; Martin et al., 2013; Kong et al., 2015; Panoli et al., 2015; Tekleyohans et al., 2017). A plethora of genetic factors have been identified to be involved in the development of embryo sac and its cellular differentiation (Yang et al., 2010; Chevalier et al., 2011; Erbasol Serbes et al., 2019). In the following paragraphs, specific genetic regulations of the central cell are summarized (Table 1).

**TABLE 1:** Summary of genes involved in central cell function and specification.

Gene name	Gene description	Function in FG/Phenotype	Molecular machinery/Cellular process
AGL80	MADS box protein	Central cell switch to accessory cells	Transcription regulation
AGL61	MADS box protein	Central cell switch to accessory cells, central cell degeneration	
TPL	Transcription co-repressor	Central cell specification	
CCG	TFIIB family	Central cell-mediated pollen tube attraction/Failed pollen tube attraction	
CBP1	Components of transcription complex		
GFA2	Homolog of yeast Mdj1p, chaperone	Failed synergid cell death and polar nuclei fusion	Mitochondria function
AAC2	ATP/ADP translocator	Unfused polar nuclei, persistent antipodal cells and reduced egg cell size	
SYCO/FIONA	Cysteinyl t-RNA synthetase	Life span of the antipodal cells, failed polar nuclei fusion	
GCD1	A conserved mitochondrial protein	Unfused polar nuclei, smaller egg cell	
CLO	Spliceosomal components	Unfused polar nuclei, switch of the synergids and central cells to the fate of egg cells	RNA processing
LIS			
ATO			
MAA3	RNA helicase	Unfused polar nuclei, pollen tube attraction	
Bip1/2	ER chaperones	Unfused polar nuclei	ER homeostasis
ERdj3A/B			
P58IPK			
SEC22	SNARE protein	Unfused polar nuclei	Membrane dynamics
WYR	Ortholog of the Inner Centromere Protein (INCENP)	Central cell differentiation, additional egg cells	Chromosome regulation
DME	DNA glycosylase	Required for maternal expression of imprinted genes in the central cell	
FIS2, MEA, FIE	PRC2 complex	Inhibit cell autonomous endosperm development	
CKI1	Histidine kinase	Cell fate of central cell and antipodal cells failed polar nuclei fusion	Cytokinin signaling pathway

## Central Cell Development and Specification

In contrast to other gametophytic cells, the central cell is characterized by its central position and large volume, including a large central vacuole at the chalazal end, two polar nuclei, cytoplasm and organelles that are inherited from the developing syncytial gametophyte. The polar nuclei fuse before fertilization in Arabidopsis, but after fertilization in cereals (Mol et al., 1994). Hence, the central cell is homodiploid (2n) in most angiosperms and polyploidy in some other species due to fusion of more than two nuclei, but remains haploid in some basal angiosperms such as Nymphaeales and Austrobaileyales (Figure 1B). The variation of the ploidy of the central cell can impact the paternal-to-maternal genome ratio that contributes to the seed size. Although central cell is the hallmark of angiosperms and prerequisite for double fertilization, molecular mechanisms controlling its development and specification are poorly understood (Berger, 2003; Yang et al., 2010).

### Formation of Central Cell

Functional dissection of several genetic factors has shed light on the molecular mechanism of central cell formation. After cellularization, the two polar nuclei fuse to give rise to a large central cell nucleus, and defect of nuclei fusion would affect the function and specification of central cell. Mitochondria play an active role in polar nuclei fusion. *GAMETOPHYTIC FACTOR2* (*GFA2*) encodes a mitochondrial chaperone that is required for the outer membrane fusion of polar nuclei (Christensen et al., 2002). Loss of function of GAMETE CELL DEFECTED1 (GCD1), a conserved mitochondrial protein, causes unfused polar nuclei, and the egg cell is also smaller compared to the wild-type, indicating that GCD1 is required for the development of both female gametes (Wu et al., 2012). Mutant in mitochondrial cysteinyl t-RNA synthetase SYCO ARATH (SYCO) and *ATP/ADP translocator 2* (*AAC2*) results in unfused polar nuclei and persistent antipodal cells (Kägi et al., 2010). In addition, endoplasmic reticulum (ER) chaperones are also involved in this process. Loss of function of Arabidopsis ER chaperone genes *BiP1* and *BiP2* also causes unfused polar nuclei (Maruyama et al., 2010). BiP proteins can interact with ER-resident J-domain protein to mediate polar nuclei membrane fusion (Maruyama et al., 2014). The J-domain proteins ERdj3A and P58IPK mediate outer nuclear membrane fusion, while ERdj3B and P58IPK regulate inner nuclear membrane fusion. RNA metabolism and processing are also involved in central cell development. A homolog of yeast RNA helicase MAA3 (MAGATAMA3) is required for polar nuclei fusion and pollen tube attraction (Shimizu et al., 2008). Arabidopsis genes *LACHESIS* (*LIS*), *CLOTHO* (*CLO/GFA1*), and *ATROPOS* (*ATO*) encode core spliceosomal components and are initially expressed throughout the female gametophyte. Defect of these genes causes the switch of the synergids and central cells to the fate of egg cells (Groß-Hardt et al., 2007; Moll et al., 2008). In *lis* and *clo* mutant, polar nuclei also fail to fuse (Groß-Hardt et al., 2007; Völz et al., 2012). Furthermore, Soluble N-Ethylmaleimide-Sensitive Fusion Protein Attachment Protein Receptors (SNARE) gene *SEC22* is also involved in polar nuclei fusion (El-Kasmi et al., 2011). ROS accumulation is correlated with the activation of central cell reporter genes, thus ROS may also be involved in central cell development (Martin et al., 2013). Moreover, *wyrd* (*wyr*) mutant of the putative plant ortholog of the *Inner Centromere Protein* (*INCENP*), produces additional egg cells at the expense of the accessory synergid cells and is defective in central cell differentiation (Kirioukhova et al., 2011). These studies highlighted the roles of mitochondria, ER, membrane dynamics, and RNA processing in the maturation of the central cell (Table 1).

### CKI1 Signaling Pathway

Genetic studies demonstrated that cytokinin signaling pathway is required for female gametophyte development and specification of central cell fate (Pischke et al., 2002; Hejátko et al., 2003; Deng et al., 2010; Cheng et al., 2013; Yuan et al., 2016). Disruption of the two-component sensor histidine kinase CKI1, an activator of the cytokinin signaling pathway, causes abortion of the central vacuole and degradation of the female gametophyte after completion of three mitotic divisions (Pischke et al., 2002; Hejátko et al., 2003; Deng et al., 2010). Later using cell-specific fluorescent markers, it was found that the egg marker is misexpressed in all nuclei while the central cell marker is either shut down or misexpressed in antipodal nuclei, and with synergid marker expressed in the central cell in the syncytial *cki1* mutant embryo sac (Yuan et al., 2016). These indicate that the central cell was switched to the egg cell fate in *cki* mutant. Consistently, ectopic overexpression of *CKI1* can switch on the central cell-specific markers in the micropylar gametophytic cells (Yuan et al., 2016). These data suggest that CKI1 is required for the specification of the central cell and antipodal cells and also restriction of the egg cell fate in the central cell. Interestingly, CKI1 protein is ER-localized and initially spread all over at two-nucleate stage and later restricted to the chalazal portion of the syncytial embryo sac at eight-nucleate stage, and concentrated around the central cell nucleus in mature embryo sac (Yuan et al., 2016). This coincides with the enriched cytokinin signaling in the chalazal due to local cytokinin biosynthesis and receptor expression (Cheng et al., 2013). Nevertheless, it remains unknown whether cytokinin itself plays a direct role or not, since CKI1 can activate downstream cytokinin signaling independent of cytokinin and lacks cytokinin-binding ability (Kakimoto, 1996; Yamada et al., 2001). Additionally, the central cell expression of *IPT8* for cytokinin biosynthesis can partially rescue *cki1* female gametophyte lethal phenotype, suggesting that the activation of cytokinin receptor signaling can to some extent complement the loss of *CKI1* (Deng et al., 2010). Of note, the *cki1* mutant also shows failed polar nuclei fusion (Yuan et al., 2016; Zhang et al., 2020).

The dynamic localization of CKI1 protein also implies a role of polar nuclei movement for central cell specification. How CKI1 specifies central cell fate and the role of cytokinin remain to be investigated (Weijers, 2016). There is evidence that CKI1 acts upstream of histidine phosphotransfer proteins (AHPs), which are required for female gametophyte development as well (Deng et al., 2010; Cheng et al., 2013). AHPs are also involved in central cell and antipodal cell fate determination (Liu et al., 2017). And mutation of *MYB116*, a proposed target of CKI1 signaling, also affects female gametophyte development (Rabiger and Drews, 2013). Together, these data strongly suggest that CKI1-mediated signaling pathway plays a critical role in central cell specification.

### Transcriptional Control of Central Cell Fate

Previous studies have identified pairs of MADS-box transcription factors of the AGL family that are specifically expressed in the central cell (Bemer et al., 2010). These transcription factors often act as hetero- or homodimer to bind CArG box of target genes. Both *AGL80* and *AGL61/DIA* are expressed specifically in the central cell and can form a heterodimer. Loss of either *AGL80* or *AGL61/DIA* function impairs central cell maturation and renders central cell non-functional (Portereiko et al., 2006; Bemer et al., 2008; Steffen et al., 2008). Recently, it was reported that the central cell of *agl80* mutant ectopically expresses synergid- and antipodal-specific marker genes (Zhang et al., 2020). This indicates that AGL80-AGL61/DIA complex is required for specification of central cell fate. Except for the type I MADS-box DNA binding domain, AGL80, but AGL61/DIA, contains a transcription repression domain, the EAR motif that is essential for AGL80 function and required for its interaction with the co-repressor TOPLESS (TPL) proteins (Zhang et al., 2020), suggesting that AGL80 acts as a transcription repressor in the central cell. Recent data, indeed, showed that AGL80 represses transcription of the synergids-specific *MYB98* genes, the major determinant factor of the synergid cell fate, in the central cell by directly binding to the CArG boxes present in the upstream promoter region of *MYB98* gene (Zhang et al., 2020). Consistently, ectopic expression of *AGL80* in synergids can repress the expression of *MYB98*, and switches on the transcriptional expression of central cell-specific gene *DD22* in the synergid (Zhang et al., 2020). In addition, AGL80 can form a homodimer in Arabidopsis protoplasts. Nevertheless, it’s also possible that ectopic expression of AGL80 in the synergids can also switch on the expression of AGL61. This implies that AGL80 orchestrate gene transcription, and the EAR motif-mediated transcriptional repression plays a critical role in restricting central cell fate. It remains to be determined whether other AGLs in the central cell are also required for central cell function and the roles of the AGL80 orthologs in cereals. *AGL80* is required for the expression of *DEMETER* (*DME*), a DNA glycosylase that is required for DNA demethylation in the central cell, as well as the determination of central cell and endosperm development (Portereiko et al., 2006; Park et al., 2016). These findings suggest that AGL80 plays a critical role to orchestrate the epigenetic pathway in the central cell.

### Epigenetic Control of Central Cell

DME-mediated demethylation is required to activate the transcription of the Polycomb Repressive Complex 2 (PRC2) components in the central cell (Köhler et al., 2012). Disruption of the PRC2 complex causes simultaneous nuclei division of the central cell before fertilization and seed abortion (Köhler et al., 2012; Raissig et al., 2013; Schmidt et al., 2013). These suggest active involvement of epigenetic regulation in the maintenance of the central cell.

At epigenetic regulation level, the central cell is drastically distinct from the egg cell. The EAR motif-containing repressors suppress target gene expression through chromatin modification of regulatory regions by histone deacetylation, often via forming complex with co-repressor TPL and histone deacetylases. In central cell of Arabidopsis and rice, locus-specific and active DNA demethylation contribute to the maternal chromosome hypomethylation in the endosperm (Park et al., 2016). The repressive mark H3K9me2 was found to distinctly distribute in the egg cell, central cell and their fertilization products, suggesting epigenetic dimorphism between the two female gametes (Pillot et al., 2010). The euchromatin distributed protein LHP1/TFL2 is associated with silenced loci enriched in H3K27me3 and shows lower expression in the central cell than the egg cell (Pillot et al., 2010). It’s interesting that this dimorphism is established after embryo sac cellularization (Pillot et al., 2010). The epigenetic dimorphism is also suggested by the expression of different histone isoforms in the two female gametes (Ingouff et al., 2010). Furthermore, central cell undergoes demethylation at small nucleosome-depleted transposable elements (Ibarra et al., 2012). In addition, central cell is evidenced to be transcriptionally more active than the egg cell as shown by the immunostaining of active RNA polymerase II (Garcia-Aguilar et al., 2010). These studies suggest more active chromatin state in the central cell than the egg cell which is apparently correlated with the transcriptional activity. This epigenetic dimorphism has been proposed to be biologically significant but still waits further evidence (Baroux et al., 2011). Detailed discussion on the epigenetic regulations in the female gametophyte, including the difference between the central cell and other sister cells can be referred to other reviews (Armenta-Medina et al., 2011; Köhler et al., 2012; Kawashima and Berger, 2014; Ashapkin et al., 2019).

### RNA Processing in Central Cell Specification

Except for transcriptional regulation, RNA processing pathway also participates in central cell specification as discussed above. In *lis*, *clo*, and *ato* mutants, the central cell and egg cell identities are misspecified (Groß-Hardt et al., 2007; Moll et al., 2008; Völz et al., 2012). In *clo/*+ mutant, the mutant antipodal cells adopts central cell fate as exhibited by changed position, membrane disintegration, nuclei fusion, and transcriptional activation of central cell marker genes (Moll et al., 2008).

In summary, these studies suggest multiple-layered and complex regulation of central cell fate and full understanding of the underlying mechanism is still a long way to go.

## Cell-Cell Communication Between Central Cell and Its Neighbors

Cell-cell communication is ubiquitous in plant development and stress response. In the embryo sac, the intercellular signaling has been suggested to be critical for its development and function. The central cell directly contacts with all the sister cells in the embryo sac, which supports its extensive intercellular interaction (Figure 1C).

### Central Cell Control on the Egg

The ubiquitously expressed mitochondria-localized protein GCD1 is required for the mutual signaling between the egg and central cell (Wu et al., 2012). Expression of *GCD1* in either the egg cell or the central cell can rescue the embryo sac defect. It is reasonable to speculate that this cell-cell communication is important for the developmental coordination of the two dimorphic female gametes. The GCD1 is a conserved protein, but its molecular function in the central cell is currently unclear (Wu et al., 2012).

### Central Cell Control on Synergid

Laser ablation experiment on the central cell suggests non-cell autonomous regulation of the central cell in the full differentiation of the synergid (Susaki et al., 2015). This regulation was also evidenced by the genetic regulation of the central cell-expressed *CCG* and *CBP1* on the transcription of the synergid-expressed *MYB98* gene and pollen tube attractants (Chen et al., 2007; Li et al., 2015; Meng et al., 2019). CCG is an early transcribed gene in the central cell (Zhang et al., 2020). The expression of *CCG* in the central cell is not affected in *agl80* mutant embryo sac that fails to specify the central cell fate (Zhang et al., 2020). Recently, *MYB98* was shown to be expressed before cellularization, which raises the possibility that the cell-cell communication might be traced back to the nucleus-nucleus communication in the coenocyte (Susaki et al., 2020). It’s still unclear whether *CCG* transcription is also initiated before cellularization. The central cell determinant factor AGL80-GFP fusion protein was initially detected right before the polar nuclei fusion and *MYB98* expression in the synergids is not affected in *agl80* mutant (Portereiko et al., 2006; Zhang et al., 2020). These findings imply that the central cell-synergid intercellular communication likely initiates early during the differentiation of these cells.

### Central Cell Control on Antipodal Cells

The central cell-expressed mitochondria-localized cysteinyl t-RNA synthetase FIONA/SYCO regulates the life span of the antipodals in a non-cell autonomous manner (Kägi et al., 2010). Interestingly, targeted disruption of the electron transport chain in the central cell mitochondria revealed that the lifespan of antipodal cells is coupled to the metabolic activity of the central cell (Kägi et al., 2010). This suggests that the metabolic state of the central cell is correlated with the differentiation of the surrounding cells.

### Molecular Mechanism of Intercellular Signaling

The intense cell-to-cell communication between the four cell types could be quite necessary and intriguing as one considers the embryo sac is just a reduced “parasitic” miniature derived from individual plants in the long history. The underlining molecular mechanism of these intercellular signaling is unknown and several lines of evidence provide some clues. Several mechanisms, such as signaling through the extracellular matrix molecules and symplastic trafficking, are evidenced or proposed to be involved during female gametophyte maturation and function.

Symplastic connection is the cellular channel of intercellular transport of transcription factors, small RNAs or other small molecules and has been suggested to function in intercellular transport of small molecules within the embryo sac (Han et al., 2000; Li et al., 2015, 2018; Erdmann et al., 2017). Using fluorescent dyes, it was clearly shown that symplastic connection exists within the embryo sac. In *Torenia fournieri* embryo sacs, molecules less than 10 kDa can diffuse freely between gametophytic cells, while molecules larger than 10 kDa cannot (Han et al., 2000). More recent data showed that molecules up to 20 kDa, including 24-nucleotide small RNAs, can move between the central cells and egg cells in Arabidopsis, while this movement capacity is lost after fertilization (Erdmann et al., 2017). The small RNAs produced in the endosperm and even central cell are speculated to move to the egg and embryo to direct DNA methylation (Ibarra et al., 2012). These observations strongly suggest that material exchange and cell-cell communication exist within the embryo sac, but compelling genetic evidence and the underlining cellular support, such as the presence of plasmodesmata, for this movement still need further exploration.

Apoplastic peptides are widely employed in intercellular signaling in plants. Transcriptome profiling revealed that diverse secreted peptides are highly enriched in the embryo sac. In Arabidopsis, *ccg* mutant ovules lose the ability to attract pollen tube (Chen et al., 2007). It has been shown that in *ccg* ovules, more than a hundred of secreted peptide-encoding genes are down-regulated (Li et al., 2015). These peptides are highly expressed in the central cells and secreted to the apoplast and even to the intercellular space of the integuments (Li et al., 2015). It is possible that some protein factors required for the later endosperm development have been activated in advance in the central cell before fertilization. Three central cells-expressed cysteine-rich peptides, ESF1s, were identified to be required for the development of embryo suspensor (Costa et al., 2014). These ESF1s are also down-regulated in *ccg* ovules (Li et al., 2015). It is still a mystery whether the numerous secreted peptides down-regulated in *ccg* have roles in cell-cell communication between the central cell and its neighbors or intercellular signaling between the fertilization products. This type of regulation has been reported, for example, in maize the egg cell-expressed secreted peptide ZmEAL1 regulates the cell fate of the antipodal cells (Krohn et al., 2012). In addition, one clue for the role of exosome signaling is the detection of expression of tetraspanin family members in the embryo sac (Boavida et al., 2013), since tetraspanins in plant were shown to be required for exosome formation that is required for the release of anti-pathogen small RNAs (Cai et al., 2018). However, the function of these tetraspanins in embryo sac is still unknown as the mutants exhibit no phenotype.

### Distinction Between Central Cell and Egg Cell in Fusion With Sperms

Double fertilization entails fusion of sperm cells with the two dimorphic female gametes, the egg and the central cell. The potentiality of sperm cells for fertilization is activated by the *EGG CELL1s* (*EC1s*) that are expressed specifically in the egg cell, but not in the central cell (Márton et al., 2005). It is not known whether the central cell employs its own way to activate the sperm or takes advantage of the egg-secreted EC1. The putative receptor for gamete adhesion, GEX2, contains an extracellular domain and is required for the sperm fusion with both female gametes (Mori et al., 2014). This implies the presence of similar or the same ligand on the surface of egg and central cell. Live imaging studies showed a lagged sperm fusion with the central cell than the egg cell, indicating that differences between the two fusion events may exist (Denninger et al., 2014; Hamamura et al., 2014). Study with the polyspermic *tetraspore* (*tes*) mutant has suggested that polyspermy block exists in egg, but not in central cell (Scott et al., 2008). Other reports suggest that the egg cell can also fuse with two sperms to form triploid embryo with the central cell fuses with one sperm cell (Grossniklaus, 2017; Mao et al., 2020). Another distinction between the central cell and the egg cell is that plasma membrane fusion with sperm can trigger central cell mitotic division, suggesting signaling link between plasmogamy and activation of the nuclear division (Aw et al., 2010).

## Evolution of Central Cell

The evolutionary origin of the double fertilization that is characterized by the emergence of the central cell is still mysterious, since the double fertilization phenomenon was discovered in the late 1890s. Based on the diversity of embryo sac, different hypothesis were raised to explain the evolution of different ploidy of endosperms and the adaption significance, i.e., the origin of structural novelty and its relative fitness (Friedman et al., 2008). The developmental evolution of the embryo sac is tightly related to the endosperm genetics and the variation of endosperms will gives rise to phenotype variations that are subject to natural selection. Among the extant flowering plants, seven types of endosperms exist. But our understanding on its evolutionary trajectory, adaption and even molecular modulation is still very limited.

Genetic evidence in model plant Arabidopsis has now provided new clues on the central cell evolution. AGL80 loss-of-function mutant was recently found to be featured by the failed fusion with sperm cell and switch of marker genes to the accessory cells. The study of *AGL80* suggests a conserved mechanism of central cell determination in Brassicaceae by the EAR-motif mediated gene repression mechanism (Zhang et al., 2020). This indicates multiple divergence of central cell specification during evolution and more primitive mechanisms are to be found. It would be interesting to investigate whether AGL80 homologs in other species are also involved in central cell specification or function even if EAR motif is not present. Although the EAR motif is only conserved in Brassicaceae, whether other transcription repression mechanism executed by AGL80 homologs or other transcription factors in monocot and other taxa are still to be unveiled. From this study, it appears that the determination of central cell fate is not conserved as expected, instead it has originated more than one time. The evolutionary conservation of AGL80 in the central cell in the Brassicaceae family may reflect fast evolution of the central cell that is wired for postzygotic reproductive isolation. The incompatibility of two species in the hybrid endosperm constitutes one of the major postzygotic isolation.

**FIGURE 2:**
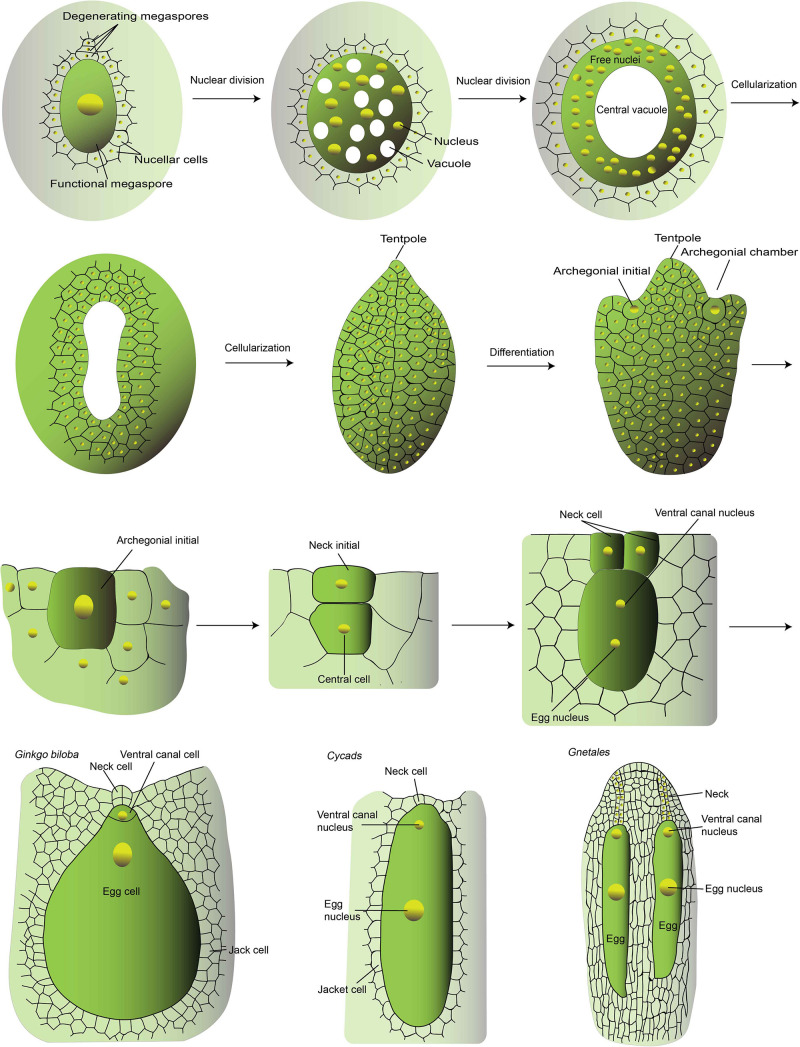
The development of Ginkgo archegonium. This is a schematic diagram of the major stages of female gametophyte development of Ginkgo and the mature female gametophyte of Cycads and Gnetales. The schematic diagram was drawn according to Dhote, Gupta and Bijoy G (www.biologydiscussion.com) and Wang et al. (2014). The female gametogenesis of different gymnosperms is quite similar. The development of Ginkgo female gametophyte is described as a representative. In brief, it develops from the large functional megaspore, the remaining spore after meiosis. The nucleus of the megaspore divides mitotically to generate thousands of free nuclei within a cell with a large central vacuole. Thereafter, the cellularization takes place in a centripetal fashion and finally the vacuole is obliterated. The cellularized gametophyte is usually called endosperm, because it undertakes the role of embryo-nourishing like the fertilization-generated endosperm in flowering plants. Then the archegonium formation initiates. Two to four cells differentiate into the archegonial initials, which then divide periclinally to form an outer small primary neck initial and a large central cell. The primary neck initials divide vertically twice resulting in four neck cells. The central cell divide asymmetrically to generate the upper ventral canal cells which disappears quickly and a large egg cell. With the expansion of the egg cell, the neck cells are pushed outside and finally degenerate to form the opening for the sperm entry. In Cycads and Gnetales, the ventral canal nucleus and the egg nucleus are within the same cell without cell wall separation. In Gnetales, a file of neck cells are formed thus generating a longer canal for sperm entry.

In gymnosperms (the non-flowering seed plants), like Cycads, Gnetales, and Gingko, the surrounding gametohpytic cells take the role of endosperm function to nourish the embryo. The female gametophyte undergoes numerous rounds of mitosis to produce a coenocytic cell with approximately thousands of free nuclei (Figure 2; Soma, 1997). At maturation, the egg-containing archegonia are structurally alike that of the moss (Figure 2), and the pollen grain contains two sperm cells at maturity and only one sperm cell would transmit the genome to the progeny. It was suggested that the gametophytic cells surrounding the archegonia function analogously to the nourishing endosperm in angiosperms. These multicellular gametophytes store reserves before fertilization to support the embryogenesis after the egg cell fertilization. With the emergence of the central cell, the embryo-nourishing role shifts from the gametophyte to the fertilized central cell, the endosperm. It confers the flowering plants several benefits and at the same time the fertilization process become more complex. The fertilization-dependent nutrients allocation to the endosperm saves energy as embryogenesis is not always a hundred percent successful. On the other hand, endosperm, containing the genome of both parents, takes a major part in the post-zygotic hybrid barrier (Lafon-Placette et al., 2017), to help maintain the identity of species by reducing gene flow.

The evolution of the endosperm in flowering plants has been discussed for years (Berger, 2003; Friedman et al., 2008). One scenario of the origin of the endosperm is a sexualized gametophytic cell and fertilization triggers the mitotic progression of the nourishing tissue. This scenario is supported by the similar pattern between the endosperm development of angiosperms and the female gametophyte of gymnosperms. In addition, the fertilization-dependent endosperm development gives rise to the possibility of parental regulation. Another scenario of endosperm origination is that an altruistic embryo finally takes the role of embryo nourishing. At the present time, however, there is still no evidence to show the evolutionary origin of the central cell, as no transitional or primordial female gametophyte structure has been found. Even in the most ancient flowering plant, Amborella, the four-cell-typed female gametophyte has already formed. Two recent studies suggested that the signaling component of cytokine, CKI1 in Arabidopsis regulate the central cell fate, and the orthologous CKI1 gene in Ginkgo, is expressed in archegonia and the precursor and surrounding tissues (Yuan et al., 2016, 2018). This study implies that at least some factors involved in female gametophyte development have been employed in the gymnosperm.

Although no fertilization-based endosperm was generated in gymnosperms, some comparison has been made between gymnosperms Gnetales and angiosperms (Friedman, 1998). In Gnetales, the binucleate sperm is carried by the pollen and released into the binucleate egg cell. It was reported that in Gnetales, the egg cell contains two nuclei, the centrally placed one as the egg nucleus, another one is the ventral canal nucleus (Figure 2). In *Welwitschia*, only the egg nucleus is fertilized since the second sperm nuclei does not enter the egg cell and degenerates (Friedman, 2015). In *Gnetum* and *Ephedra*, the two sister nuclei of the egg cell fused with the two sister sperm nuclei, respectively (Friedman, 1990). However, the coenocytic female gametophyte matures upon the pollen tube penetration and the simultaneous fertilization of the two haploid female nuclei determines the following fate of the fertilization products. But this lack of egg differentiation is unique in Gnetales and *Welwitschia* (Friedman, 1998). After fertilization, the conenocytic tissue undergoes cellularization and both fertilization products initiate embryogenesis and only one matures with the nourishment of the surrounding gametophytic tissues. Despite the developmental similarities of the mitotic sister sperm nuclei within the same pollen and sister female gametophytic nuclei, no definitive homology of the double fertilization events between Gnetales and flowering plants has been drawn. Another remaining mystery is the single fruitful fertilization at the expanse of waste of the other sperm. The emergence of two sperms likely have driven and provided the prerequisites for the origination of the second fertilization-competent cell and double fertilization. In the absence of fossil record of species with central cell ancestor, the elucidation of the evolutionary ontogeny of this specialized cell is difficult. Genetic dissection and molecular evolution study of key genes especially that with conserved roles in central cell specification would be helpful, which benefit from the release of more and more whole genome sequences of different plant taxa. High through-put transcriptome sequencing of small amount of samples would also promote the unraveling of the genetic hierarchy and evolution of female gametophyte, although the functional study would be challenging due to the difficulty in genetic transformation of these species.

## Perspective

Nowadays, we have a more comprehensive understanding of the cell specification and intercellular signaling of central cell in molecular and evolutionary aspects. The active involvement of central cell in diverse aspect of fertilization points to an emerging importance of this non-heritable female gamete. Experimental evidence is still limited for the full understanding of this mysterious cell. Although with the studies in the past two decades, the identification of the key components and their functional connectivity remains the major hurdle in understanding of central cell function and evolution. The central cell is enriched in secreted peptides, but most of them have yet to be functionally characterized as the conventional T-DNA and gene knock-down approaches are powerless in these highly redundant and sequence-diverged gene families. In addition, the relaxation of the gene silencing machinery activates the transposable elements and a large number of genes that would make the reverse genetic study laborious. With the dawn of innovative strategies in experimental techniques, such as single-cell transcriptome and epitranscriptome, gene editing technology and increase of sequenced species, we can expect a more comprehensive understanding on central cell specification, fertilization, and coordination with the surrounding cells, as well as how the central cell helps to shape the flowering plants’ overwhelming predominance on earth.

## Author Contributions

H-JL and W-CY wrote the manuscript. Both authors contributed to the article and approved the submitted version.

## Conflict of Interest

The authors declare that the research was conducted in the absence of any commercial or financial relationships that could be construed as a potential conflict of interest.
